# Comparison of microbiota profiles of fecal samples, rectal swabs and mucosal biopsies in patients with inflammatory bowel disease

**DOI:** 10.1080/29933935.2026.2644121

**Published:** 2026-03-18

**Authors:** Tessel M. van Rossen, Kim van den Hoek, Thomas Groot, Rob H. Creemers, Maysa L. M. van Doorn-Schepens, Emma M. van Andel, Matthijs E. Grasman, Nanne K. H. de Boer, Gerd Bouma, Andries E. Budding, Paul H. M. Savelkoul, Adriaan A. van Bodegraven

**Affiliations:** a Department of Medical Microbiology & Infection Control, Amsterdam UMC, Vrije Universiteit Amsterdam, Amsterdam, The Netherlands; b Amsterdam Institute for Infection and Immunity, Amsterdam, The Netherlands; c Department of Gastroenterology and Hepatology, Amsterdam UMC, Vrije Universiteit Amsterdam, Amsterdam, The Netherlands; d Amsterdam Gastroenterology Endocrinology Metabolism Institute, Amsterdam, The Netherlands; e Inbiome, Amsterdam, The Netherlands; f Department of Gastroenterology & Hepatology, Zuyderland Medical Centre, Sittard, the Netherlands; g Department of Medical Microbiology, OLVG Laboratory, Amsterdam, The Netherlands; h Department of Gastroenterology & Hepatology, Northwest Clinics, Alkmaar, The Netherlands; i Department of Medical Microbiology & Infection Control,Maastricht UMC+, Maastricht, The Netherlands; j Maastricht UMC+, Department of Gastroenterology and Hepatology, Maastricht, The Netherlands

**Keywords:** Microbiota composition, microbiota profiling, sampling methods, sample types, inflammatory bowel disease

## Abstract

Gut microbiota changes are associated with inflammatory bowel disease (IBD). In most microbiota studies, intestinal microbiota composition is examined in fecal samples, but their representativeness of mucosa-associated microbiota at inflammation sites remains unclear. This study aimed to explore microbiota composition in different IBD sample types and assess their interchangeability for research and clinical use. Multicentre, prospective, observational study including 200 IBD patients (518 samples). We compared microbiota profiles from faeces, rectal swabs and mucosal biopsies from different colon sites. Microbiota composition was analyzed by Molecular Culture™, a bacterial profiling technique based on species-specific differences in the 16S-23S interspace region of bacterial ribosomal DNA, with taxonomic classification by phylum-specific fluorescent PCR primers. Fecal samples and rectal swabs contained a higher microbial diversity and abundance than colonic biopsies. Microbiota compositions of different sample types within an individual happened to be quite dissimilar (median cosine similarities 0.43–0.53). Within individuals, biopsies from the same location in the colon were just as similar as biopsies from different locations (median cosine similarities 0.85 vs. 0.82, respectively). Fecal samples, rectal swabs and colonic biopsies of an individual show distinct microbiota profiles and should not be used interchangeably in microbiota studies or clinical applications.

## Introduction

The intestine contains more than 1000 different bacterial species, of which more than 99% belong to four phyla: Firmicutes, Bacteroidetes, Proteobacteria, and Actinobacteria.^1^^,^^2^ A “healthy” gut microbiota protects against pathogens and positively affects the human metabolism and the immune system.^2^ A disrupted intestinal microbiota, known as dysbiosis, can alter the function of the immune system. Distinct bacterial features have been described for several neurodegenerative, cardiovascular, metabolic, and gastrointestinal diseases, including inflammatory bowel disease (IBD).^3–5^

IBD is characterized by a chronic and relapsing, remitting inflammation primarily expressed in the intestine. The main subtypes of IBD are ulcerative colitis (UC) and Crohn's disease (CD). UC causes superficial mucosal inflammation, which generally originates at the anal verge and can extend in a continuous, circumferential pattern to more proximal segments of the colon.^6^ Contrary to UC, CD may involve any part of the gastrointestinal tract in a discontinuous, “segmental” pattern.^7^ The etiology of IBD remains unclear, but genetic and environmental factors, immune response dysregulation, and the gut microbiota composition play a (interconnected) role in pathogenesis.^2^^,^^8^

In most microbiota studies on IBD, fecal samples are collected to examine the intestinal microbiota composition. In some studies, rectal swabs have been used. These can be easily obtained during outpatient clinic visits and stored immediately in a standardized manner. However, it is undecided whether fecal samples and rectal swabs are representative of the mucosa-associated microbiota, let alone at the site of inflammation, in IBD patients. Studies in which the microbiota composition in these different sample types were compared are either small and sparse, or have been carried out in children, healthy subjects or patients with disorders other than IBD.^4,9–13^ This thwarts interpretation and comparison of the microbiota reports which have been published up till now.

Therefore, in this study, we compared the microbiota profiles of fecal samples, rectal swabs and colonic mucosal biopsies in a large population of 200 IBD patients (518 unique samples). Additionally, we examined biopsies derived from different colon sites. Microbiota composition was assessed by IS-pro, which is an efficient, informative and relatively quick method to study microbial communities for clinical applications with results comparable to those obtained by 16S sequencing.^14–19^ The aim of this study was to obtain more insight into the microbiota composition in different sample types in IBD patients, and to investigate whether different sample types within an individual could be used interchangeably for microbiota studies and clinical applications. In that case, any sample type (within a patient) could be used to monitor changes in microbiota composition over time, for example to assess whether the microbiota correlates with IBD-related disease activity or treatment response.

We hypothesize that mucosal biopsies differ the most from fecal samples and rectal swabs, primarily due to the sampling of mucosal versus luminal microbiota, as well as the fact that mucosal biopsies are collected after bowel cleansing. Additionally, fecal samples may offer a more comprehensive representation of the intestinal microbiota, as they reflect microbial communities from the entire gastrointestinal tract, in contrast to rectal swabs.

## Materials and methods

### Study design and participants

For this research project, data and samples from the MICROBE study cohort were used (manuscript in preparation). This was a multicentre, prospective, observational study on the gut microbiota in patients with IBD. Participating centers were one university medical center and three large teaching hospitals in the Netherlands. Participants were adult patients with IBD who had clinical symptoms of an exacerbation and who were scheduled for endoscopy. At baseline, and after 3, 6 and 12 months, fecal samples, rectal swabs and (only at baseline) colonic mucosal biopsies were collected. IBD type was determined based on a combination of clinical, biochemical, fecal, endoscopic, imaging, and histological investigations.^20^ Disease activity at baseline was determined by the endoscopic Mayo score (UC), simple endoscopic score (CD), and at follow-up visits by fecal calprotectin levels. An exacerbation was defined as an endoscopic Mayo score of ≥2 for UC patients, and a Simple Endoscopic Score for CD (SES-CD) of ≥4 for CD patients with isolated ileal disease (Montreal classification L1) and a SES-CD of ≥7 for CD patients with (ileo)colonic disease (Montreal classification L2/L3).^21^^,^^22^ For this current project, only data and samples collected at baseline were used. Since we used samples from the MICROBE study, a convenience sampling approach was employed, and no formal power calculation was conducted for this explorative study. Nevertheless, previous studies comparing microbiota composition across different sample types have used sample sizes ranging from 10 to 83 patients.^4,9–13^ Additionally, a post hoc power analysis for the outcome cosine similarity between samples was conducted. To detect a mean difference of 0.2 between groups, assuming a standard deviation of 0.4 with a two-sided *α* of 0.05 and 80% power, 34 pairs (68 samples) were required. Based on this, we anticipate that our sample size of 200 patients would be sufficiently large to yield reliable results.

This study was approved by the Medical Research Ethics Committee of the Amsterdam University Medical Center, formerly the VU University Medical Center. Written informed consent was obtained from all the subjects prior to participation. The study was carried out in one university medical center and four large teaching hospitals in the Netherlands. The participants were adult patients with UC or CD who had clinical symptoms of IBD exacerbation and underwent endoscopy. The diagnosis of IBD had to be established for at least six months. The exclusion criteria were the use of any antibiotic in the month prior to inclusion or a history of extensive surgery for CD or UC.

Before bowel preparation, a fecal sample and rectal swab were collected. In all patients, during colonoscopy two biopsies were derived approximately 20 cm from the anal verge, and in Crohn's disease patients also from the most inflamed bowel segment and/or an ulceration (if present).

### Sample handling

A fecal sample was collected by the patient with the use of a feces collection sheet attached to the toilet seat (www.fecesvanger.nl) and transferred into a sterile container. For rectal swabs, a FLOQSwab (Copan, Murrieta, USA) was inserted about 2 cm into the anal canal and gently rotated for two seconds. Rectal swabs were collected by the patient or a trained researcher. Afterwards, the swab was placed into an Eppendorf tube containing 1 ml standard reduced transport fluid (RTF) buffer.^23^ All samples were stored at −20 °C within 1 h after collection. Mucosal biopsy specimens collected during endoscopy were placed in empty and sterile Eppendorf tubes, immediately snap frozen in liquid nitrogen and stored at −20 °C, subsequently.

### Microbiota and data analysis

DNA was isolated according to the instructions by the manufacturer and by using the NucliSENS easyMAG automated DNA isolation machine and reagents (Biomérieux, Marcy l'Etoile, France). After isolation, the DNA was stored at 2–8 °C. The specific A protocol was selected for all sample types. For the fecal samples and rectal swabs, a 110 μl eluate volume and for mucosal biopsy specimens 70 μl eluate volume was selected. Isolated DNA was stored at 2–8 °C.

Microbiota analysis was performed by using the IS-pro technique, using the Molecular Culture Microbiota® kit (inbiome, Amsterdam, The Netherlands) according to the manufacturer's instructions. An internal positive control per sample, and multiple positive controls per PCR batch, were present in each PCR amplification to detect potential inhibition. Negative controls were added to detect potential contamination. In case of PCR inhibition, DNA was diluted 1:5 and 1:10 and the PCR was repeated. Samples not passing the quality control (not passing our validated cut-off for PCR inhibition) were excluded. IS-pro is a eubacterial profiling method, based on bacterial species-specific differences in the length and number of the 16S-23S interspace (IS) regions of the ribosomal DNA, with taxonomic classification by phylum-specific fluorescent labeling of PCR primers.^24^ This results in a microbiota profile (see Figure S1 for an example). The peaks within the same color identify the specific species within that phylum. The position (nucleotide length) of each peak corresponds to a specific bacterial species with a certain intensity (abundance) in relative fluorescent units (RFU). The IS-pro technique is an effective method to study microbial communities for clinical applications.^14–18^ DNA fragments were visualized with the Spotfire software package (TIBCO, Palo Alto, CA, USA). Microbial abundance and Shannon diversity index were calculated per phylum: Bacteroidetes, Proteobacteria or the Firmicutes, Actinobacteria, Fusobacteria, Verrucomicrobia group (FAFV).

To test for differences in microbiota compositions between samples, permutational multivariate analysis of variance (PERMANOVA) and analysis of similarities (ANOSIM) were performed based on a cosine distance matrix with 999 permutations and visualized in principal coordinate analysis (PCoA) plots. In addition, similarity of microbiota profiles on bacterial species level between samples (both between individuals as well as within individuals,) was assessed by cosine similarity. The cosine similarity of two samples was calculated by vectorizing the bacterial abundances, represented as decimal numbers, and calculating the cosine angle between the two one-dimensional vectors. This metric can range from 0 (completely different microbiota profiles) to 1 (identical profiles).

**Figure 1.**
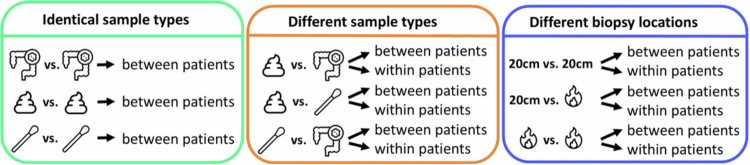
Study design. *20 cm: biopsy 20 cm ab ano; fire icon: biopsy from most diseased bowel segment or an ulcer.*

First, samples of the same sample type (colonic biopsy 20 cm from the anal verge, feces or rectal swab) were compared between patients. For each patient, the microbiota profile of one sample was compared to the microbiota profile of a sample of another random patient (three comparisons, Figure 1). Secondly, we compared microbiota profiles of different sample types between and within individuals (six comparisons, Figure 1). Next, we compared microbiota profiles of mucosal biopsies derived 20 cm proximal to the anal verge (20 cm *ab ano*) and/or from inflamed mucosa, between and within individuals (also six comparisons, Figure 1). For the *between-individual* comparisons, for each patient, the microbiota profile of one sample was compared to that of a sample from another random patient by calculating the cosine similarity between the two samples. For the *within-individual* comparison, for each patient, the microbiota profile of one sample was compared to the microbiota profile of another sample from the same patient. The results of all comparisons were visualized in boxplots, indicating the median cosine similarity (horizontal center line in the box), 25th–75th percentiles of the dataset (the box), 5%–95% percentiles (vertical bars) and outliers (dots). Differences in microbial abundance and diversity between sample types, and between similarities were evaluated using two-tailed t-test. A *p*-value of <0.05 was considered statistically significant. No correction for multiple testing was applied, given the exploratory nature of the study and the fact that all analyzes involved comparisons between two independent groups.

## Results

### Patients and samples

Two-hundred patients were enrolled in the study: 125 patients with UC, 73 patients with CD and 2 patients with IBD-U (unclassified), see Table 1. In total, 518 samples were collected for microbiota analysis from these patients: 175 fecal samples, 153 rectal swabs and 190 colonic biopsies.

**Table 1.** Patient characteristics.

Characteristics	*n* (%)
Number of patients Age in years, mean [SD] Female gender Disease characteristics: *Ulcerative colitis* *Extent of ulcerative colitis* *E1: Ulcerative proctitis* *E2: Left-sided UC (distal tot splenic flexure)* *E3: Extensive UC (pancolitis)* *Crohn's disease* *Location of Crohn's disease* *L1: ileal* *L2: colonic* *L3: ileocolonic* *L4: isolated upper digestive* *IBD-U* Active disease^#^	200 (100) 48 [17] 113 (57) 125 (62) n = 124* (100) *34 (27)* *57 (46)* *33 (27)* *73 (37)* *n = 73 (100)* *18 (25)* *22 (30)* *33 (45)* *4 (5)* *2 (1)* 128 (64)

^*^ *Data on extent of disease of one UC patient was missing.*

^#^ *Defined as an endoscopic Mayo score ≥2 for UC and IBD-U patients, and a simple endoscopic score for Crohn's disease (SES-CD) ≥4 for CD patients with isolated ileal disease and an SES-CD ≥7 for CD patients with (ileo)colonic disease.*

### Microbiota composition in different sample types

**Figure 2.**
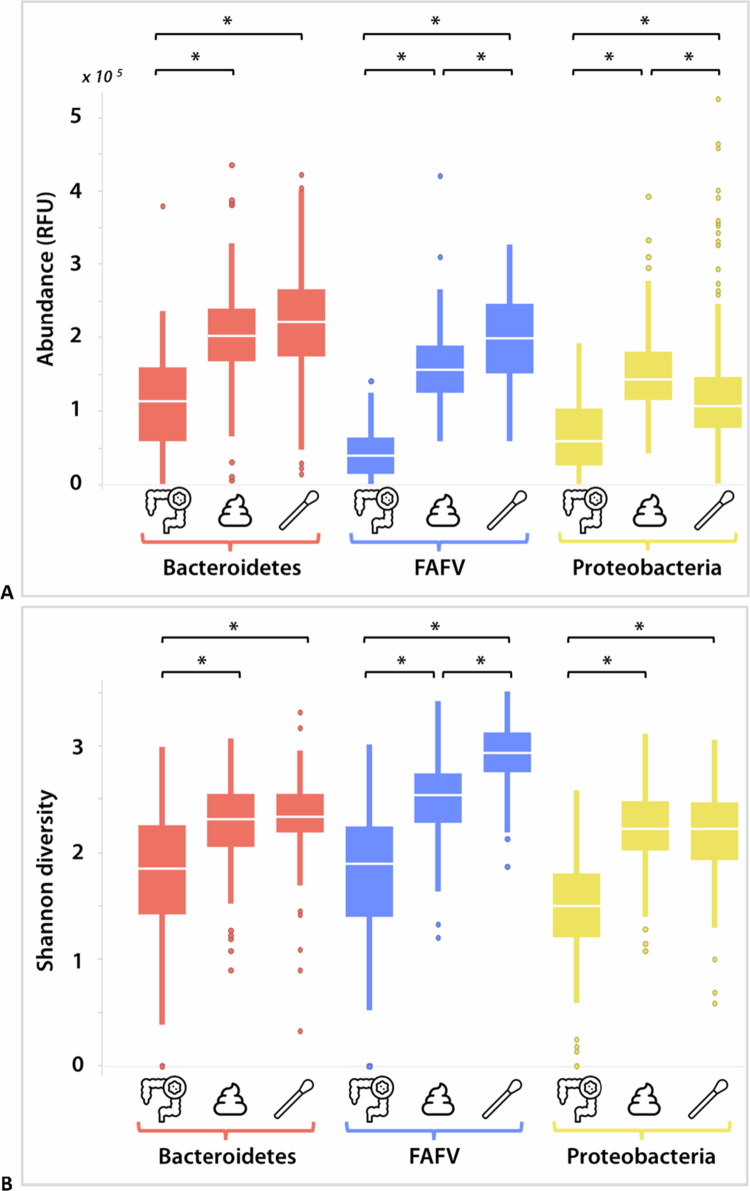
Microbial abundance (A) and diversity (B) per phylum in colonic biopsies (*n *= 190), fecal samples (*n *= 175) and rectal swabs (*n *= 153). Only statistically significant differences within the same phylum are indicated (*p < 0.05). *FAFV: Firmicutes, Actinobacteria, Fusobacteria, Verrucomicrobia; RFU: relative fluorescent units.*

The bacterial abundance and Shannon diversity per phylum for each sampling type are shown in Figure 2. For all the phyla, the abundance and diversity of the fecal samples and rectal swabs exceeded those of the colonic biopsies. Furthermore, rectal swabs had a higher abundance and diversity of Firmicutes, Actinobacteria, Fusobacteria and Verrucomicrobia (FAFV) than did fecal samples. In contrast, the abundance (but not the diversity) of Proteobacteria was higher in the fecal samples compared to rectal swabs.

The fifteen most abundant bacterial species per sample type are shown in Table 2. *Phocaeicola vulgatus* was the most abundant species in all sample types*. Streptococcus bovis, Escherichia coli, Bacteroides fragilis* and *Prevotella intermedia* were also highly abundant. *Staphylococcus lugdunensis* was also abundant in rectal swabs and feces but not in colonic biopsies. In all sample types, strictly (as well as facultative) anaerobes were highly abundant.

**Table 2.** Top-15 most abundant bacterial species in colonic mucosal biopsies, fecal samples and rectal swabs.

Colonic biopsies		Fecal samples		Rectal swabs	
***Species (relative abundance)***		***Species (relative abundance)***		***Species (relative abundance)***	
*Phocaeicola vulgatus Streptococcus bovis* *Escherichia coli* *Bacteroides fragilis* *Prevotella intermedia* *Enterococcus gallinarum* *Bacteroides distasonis* *Klebsiella pneumoniae* *Moraxella nonliquefaciens* *Clostridium innocuum* *Cutibacterium granulosum* *Clostridium sordelii* *Shigella sonnei* *Atopobium vaginae* *Lactobacillus paracasei*	*0,27* *0,23* *0,10* *0,08* *0,03* *0,03* *0,03* *0,02* *0,02* *0,02* *0,02* *0,02* *0,02* *0,01* *0,01*	*Phocaeicola vulgatus* *Streptococcus bovis* *Bacteroides fragilis* *Escherichia coli* *Prevotella intermedia* *Enterococcus gallinarum* *Granulicatella adiacens* *Staphylococcus lugdunensis* *Pseudomonas putida* *Clostridium tetani* *Stenotrophomonas maltophilia* *Peptostreptococcus anaerobius* *Streptococcus intermedius* *Citrobacter koseri* *Bacteroides distasonis*	*0,18* *0,15* *0,10* *0,04* *0,04* *0,03* *0,03* *0,03* *0,03* *0,02* *0,02* *0,02* *0,02* *0,01* *0,01*	*Phocaeicola vulgatus Bacteroides fragilis group* *Streptococcus bovis* *Staphylococcus lugdunensis* *Prevotella intermedia* *Clostridium sordelii* *Peptostreptococcus anaerobius* *Finegoldia magna* *Enterobacter cloacae* *Escherichia coli* *Capnocytophaga sputigena* *Enterococcus gallinarum* *Lactobacillus iners* *Prevotella denticola* *Aeromonas sobria*	*0,16* *0,11* *0,11* *0,05* *0,04* *0,02* *0,02* *0,02* *0,02* *0,02* *0,02* *0,02* *0,02* *0,02* *0,01*

### Correlation between identical sample types from different patients

**Figure 3.**
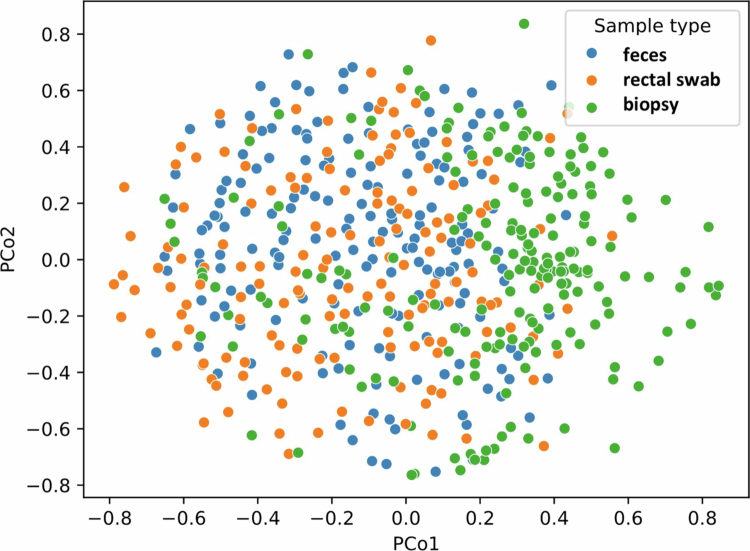
Principal coordinate analysis (PCoA) plot showing the relationship between colonic biopsies, *n *= 190, fecal samples, *n *= 175, or rectal swabs, *n *= 153, between patients. Each dot corresponds to the unique microbiota composition of a single sample. The distance between dots represents the degree of variability of microbiota composition among samples.

Samples from the same sampling mode (feces, rectal swabs or colonic biopsies) were compared between patients. For each patient, the microbiota profile (at the bacterial species level) of one sample was compared to the microbiota profile of another random patient. PERMANOVA and ANOSIM revealed no statistically significant differences among the three sample types (pseudo-F = 0.03–0,11, *p *= 0.76–0.90 and R = −0.03–0.01, *p *= 0.25–0.99, respectively). This is visualized in the PCoA plot, which shows a weak separation between different sample types (Figure 3). Furthermore, the cosine similarity between microbiota profiles of different patients was generally low, regardless of sample type (with median cosine similarities of biopsies 0.31 [IQR = 0.30–0.35], feces 0.31 [0.29–0.33] and rectal swabs 0.28 [0.27–0.29]) (Figure S2 and Table S1).

### Correlation between different sample types within- and between-individuals

**Figure 4.**
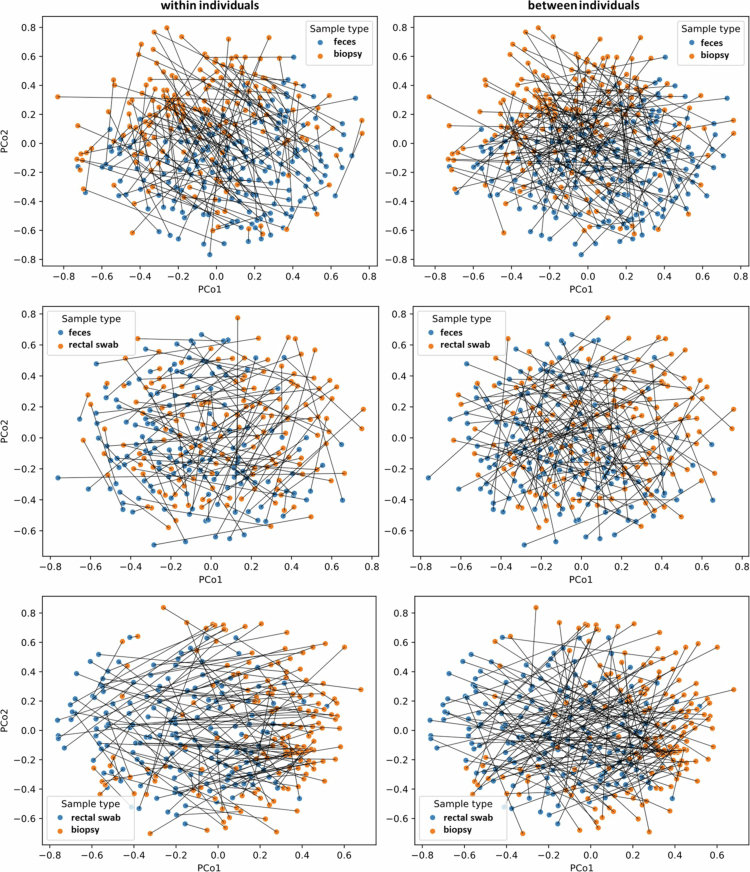
PCoA plots showing the relationship between different sample types within and between individuals. Each dot corresponds to the microbiota composition of a single sample. The distance between dots represents the degree of variability of microbiota composition among samples. Lines indicate comparisons between samples.

To assess the similarity between different sample types within individuals, for each patient, the microbiota profile of one sample type was compared to the microbiota profile of another sample type from the same patient: feces vs. biopsy (*n *= 154), feces vs. rectal swab (*n *= 136) and rectal swab vs. biopsy (*n *= 135). The large distances and long connecting lines between samples in the PCoA plots indicate that the correlation between (all three) different sample types within an individual was low (Figure 4, *within individuals* plots). The calculation of cosine similarities confirmed that the microbiota compositions of different sample types within an individual were quite dissimilar, with median cosine similarities ranging between 0.43 and 0.53 (Figure S3 and Table S1). The highest correlation was found between the microbiota profiles of feces and rectal swabs of the same patient (median cosine similarity 0.53 [IQR = 0.39–0.66]), and this was statistically significantly higher than the correlation between feces and biopsies (0.43 [0.32–0.55]), and the correlation between rectal swabs and biopsies (0.45 [0.29–0.59]).

To put these numbers in perspective, we also assessed the similarity between different sample types between individuals. Therefore, for each patient, the microbiota profile of one sample was compared to a sample from another random patient, resulting in three comparisons: feces vs. biopsy (*n *= 167), feces vs. rectal swab (*n *= 150) and rectal swab vs. biopsy (*n *= 146). As expected, microbiota compositions of different sample types between individuals were even more dissimilar than different samples within individuals (pseudo-F = 21.7–25.1, *p *= 0.001 and R = 0.06–0.07, *p *= 0.001), with median cosine similarities ranging between 0.27 and 0.30. Although subtle, in the PCoA plots the *between patients* comparisons show longer connecting lines that frequently intersect (meaning longer distances/more dissimilarity between samples), whereas within-patients comparisons show shorter lines with fewer intersections (Figure 4).

Stratified analyzes were performed to determine whether IBD type and disease activity served as confounding factors (Table S2). No statistically significant differences were observed between these subgroups. However, a trend was observed toward greater similarity between fecal and rectal swab microbiota compositions within individuals with Crohn's disease (CD) (median cosine similarity 0.59, IQR [0.44–0.71]) compared to those with ulcerative colitis (UC) (0.49, IQR [0.37–0.64]; *p *= 0.08).

### Correlation between different biopsy locations

Next, we compared microbiota profiles of mucosal biopsies derived from different or the same colon sites. In all patients, two biopsies were derived 20 cm from the anal verge and (if present, and only in Crohn's disease patients) a biopsy from the most inflamed bowel segment and/or an ulceration. For the *within-individuals* comparison, for each patient, the microbiota profile of one biopsy was compared to the microbiota profile of another biopsy from the same patient: 20  cm *ab ano* vs. 20 cm *ab ano* (*n *= 159), 20 cm *ab ano* vs. inflamed mucosa (*n *= 45), and inflamed mucosa vs. inflamed mucosa (*n *= 29). For the *between-individuals* comparison, for each patient, the microbiota profile of one biopsy was compared to a biopsy from another random patient, resulting in three comparisons: 20 cm *ab ano* vs. 20 cm *ab ano* (*n *= 167), 20 cm *ab ano* vs. inflamed mucosa (*n *= 46), and inflamed mucosa vs. inflamed mucosa (*n *= 45).

**Figure 5.**
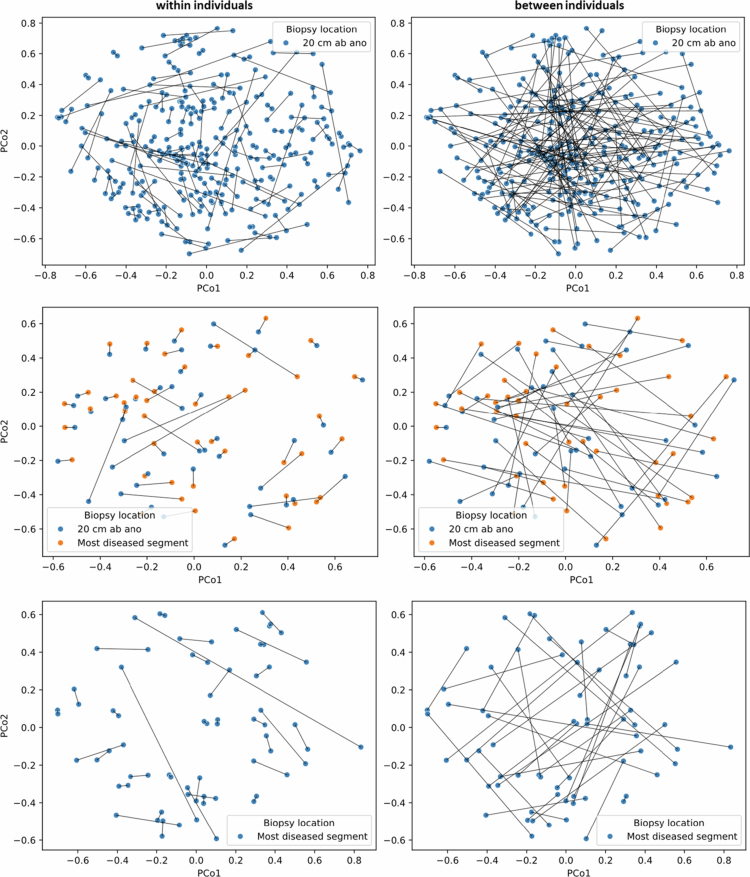
PCoA plots showing the relationship between biopsies from the same or different colon sites (20 cm *ab ano* and/or from the most diseased colon segment) within and between individuals. Each dot corresponds to the microbiota composition of a single sample. The distance between dots represents the degree of variability of microbiota composition among samples. Lines indicate comparisons between samples.

The PCoA plots clearly show that biopsies from the same patient were highly similar, as can be concluded by the short connecting lines between biopsies within individuals, regardless of biopsy location (median cosine similarities 0.82–0.85) (Figure 5, Figure S4 and Table S1). In contrast, the PCoA plots show a low correlation between microbiota profiles of biopsies from different patients (median cosine similarities 0.22–0.35). The microbiota profiles of biopsies within individuals were significantly more similar than those of biopsies between patients (pseudo-F = 48.6–181.7, *p *= 0.001 and R = 0.4–0.5, *p *= 0.001).

## Discussion

The aim of this study was to compare the microbiota profiles of fecal samples, rectal swabs and mucosal biopsies, to gain more insight into the microbiota composition in different sample types and to assess whether these sampling methods can be used interchangeably within individuals. In this study, we found that fecal samples and rectal swabs contained a higher microbial diversity and abundance than colonic biopsies. Microbiota compositions of different sample types within an individual were quite dissimilar (median cosine similarities 0.43–0.53). Within individuals, biopsies from the same location in the colon were just as similar as biopsies from different locations (median cosine similarities 0.85 vs. 0.82, respectively).

To the best of our knowledge, this work represents the largest study to date on the comparison of microbiota composition in fecal samples, rectal swabs, and mucosal biopsies from IBD patients. Our observation that bacterial diversity and abundance were higher in fecal samples and rectal swabs than in colonic biopsies was confirmed by several other studies.^9^^,^^11^ However, in contrast to our findings, in two other studies no difference in diversity between fecal samples and rectal swabs was shown, possibly due to the different populations at interest (Intensive Care Unit patients and healthy individuals instead of IBD patients).^12^^,^^13^ In agreement with previous work, we observed that the *intra-individual* correlation of different sample types was much higher than the *inter-individual* correlation.^11^^,^^25^ Furthermore, we corroborated previous observations that rectal swabs and fecal samples from the same patient are most similar, and that mucosal biopsies from different locations in the colon have similar microbiota profiles.^4^^,^^10^^,^^11^^,^^13^^,^^26^

The advantages of collecting rectal swabs for microbiota analysis is that these can be obtained in a standardized manner, concurrently to a visit to the outpatient clinic. This is in contrast with fecal samples, which patients have to collect and store at home, ideally in a freezer at −20 °C. Those fecal samples also have to be transported to the hospital or laboratory, while being kept frozen. For collection of mucosal biopsies, endoscopy is needed, which is a burden for patients. Furthermore, endoscopic procedures are generally costly. In addition, niche analysis of the colonic microbiota by biopsy sampling might be thwarted by type 1 errors such as pre-endoscopy bowel lavage and use of biopsy samplers via non-DNA-sterile biopsy ports, although the importance of the latter has been downgraded, particularly in individuals without colonic inflammation, when specific biopsy harvesting techniques are being applied.^26^

Although qualitatively different, our finding that rectal swabs and fecal samples had a higher abundance and diversity than mucosal biopsies, may be due to the sampling of the luminal versus mucosal microbiota, and because mucosal biopsies were harvested after bowel cleansing (without which focused mucosal sampling is methodologically almost impossible).^27^ We also hypothesized that feces might provide a more complete representation of the intestinal microbiota (due to the nature of sampling) than rectal swabs, but no differences in total bacterial abundance or diversity were found between these two sample types. Remarkably, in rectal swabs the FAFV abundance and diversity were higher, possibly because rectal swabs contain both luminal and mucosal bacteria, while fecal samples contain mainly luminal bacteria. Proteobacteria abundance, but not diversity, was higher in fecal samples than in rectal swabs. This was explained by the higher abundance of *E. coli* in fecal samples compared to rectal swabs. Furthermore, the top-15 of most abundant species in fecal samples also contained the Proteobacteria *P. putida*, S*. maltophilia* and *C. koseri*, while concerning Proteobacteria, the top-15 in rectal swabs (besides *E. coli*) only comprised the less abundant *E. cloacae and A. sobria*.

Interestingly, we found that biopsies derived from different locations of the intestinal mucosa of CD patients (20 cm proximal of the anal verge or from the most inflamed bowel segment) were highly similar. This suggests that a recto(sigmoido)scopy, instead of a complete ileocolonoscopy, may be sufficient to representatively sample the mucosal microbiota of CD patients, thereby reducing patients' burden and costs.

This study had several strengths, one of which was the large sample size (200 patients and 518 samples). Furthermore, we compared three different sample types/sampling methods that are reproducible and widely used in microbiota studies. Another advantage was the use of the IS-pro technique for microbiota analysis. IS-pro has been proven to be an efficient, informative and relatively swift method to study (gut) microbial communities with results comparable to those obtained by (more laborious) 16S sequencing or metagenomics.^14–19^ Since the results of IS-pro can be obtained within a day, this technique is well-suited for clinical applications. Limitations of the IS-pro technique are that some bacteria cannot be identified up to species level, and that it might overestimate the abundance of Proteobacteria as compared to more commonly applied techniques such as 16S-sequencing.^28^ Therefore, our finding that Proteobacteria abundance was higher in feces than in rectal swabs, may not have been detected by 16S-sequencing. However, which method should be considered the gold standard remains unknown.

A limitation of our study was that we did not succeed to collect all three sample types in all patients, reducing the number of samples available for analysis. This was due to logistic reasons (no liquid nitrogen or no study investigator available at day of colonoscopy), patients preferences (not willing to collect or store a fecal sample or rectal swab), or storage errors (biopsies in formalin instead of an empty Eppendorf tube, fecal samples or rectal swabs stored in the fridge instead of freezer at patients homes). Another limitation was that rectal swabs were collected partly at home and partly in hospital setting, though, previous work did not determine relevant differences in microbiota profiles between those self-sampled at home or the ones harvested by a medical professional at the outpatient clinic.^11^ Other factors that might have influenced our results are possible batch effects and inter-center variation, although several quality controls were used to reduce this (see Methods).

Several factors, such as diet and medication, can influence microbiota composition. However, the primary objective of our study was to examine potential differences between sample types within individual patients. We anticipate that medication use and diet exert a similar effect across all sample types within the same patient, and are therefore unlikely to act as relevant confounders in these within-individual comparisons. Conversely, when comparing samples between different individuals, these factors may have contributed to increased variability in microbiota composition. Since 90% of our patients did not adhere to a specific diet, thus eating standard Western (Dutch) food, subgroup analyzes based on diet were not performed. Unfortunately, information regarding medication use was unavailable, preventing us from adjusting for this potential confounder, particularly when performing comparisons between individuals. In conclusion, while it is well established that intra-individual microbiota profiles are more similar than those between individuals, our findings show that fecal samples, rectal swabs, and especially colonic biopsies differ significantly. This highlights that these sample types are not interchangeable in microbiota research or clinical applications.

## Supplementary Material

Appendices SAMPLE study revision 260225 without track changes.docx Appendices SAMPLE study revision 260225 without track changes.docx

## Data Availability

The datasets generated and/or analyzed during the current study are available in the Figshare repository, https://doi.org/10.6084/m9.figshare.31431292.
