# Supplementary material for: Comparison of microbiota profiles of fecal samples, rectal swabs and mucosal biopsies in patients with inflammatory bowel disease
Source: Gut Microbes Rep. 2026 Mar 18;3(1):2644121. doi: 10.1080/29933935.2026.2644121 (PMC13034629; doi:10.1080/29933935.2026.2644121)
Supplement: Appendices SAMPLE study revision 260225 without track changes.docx [file KGMR_A_2644121_SM6698.docx]

# Appendices


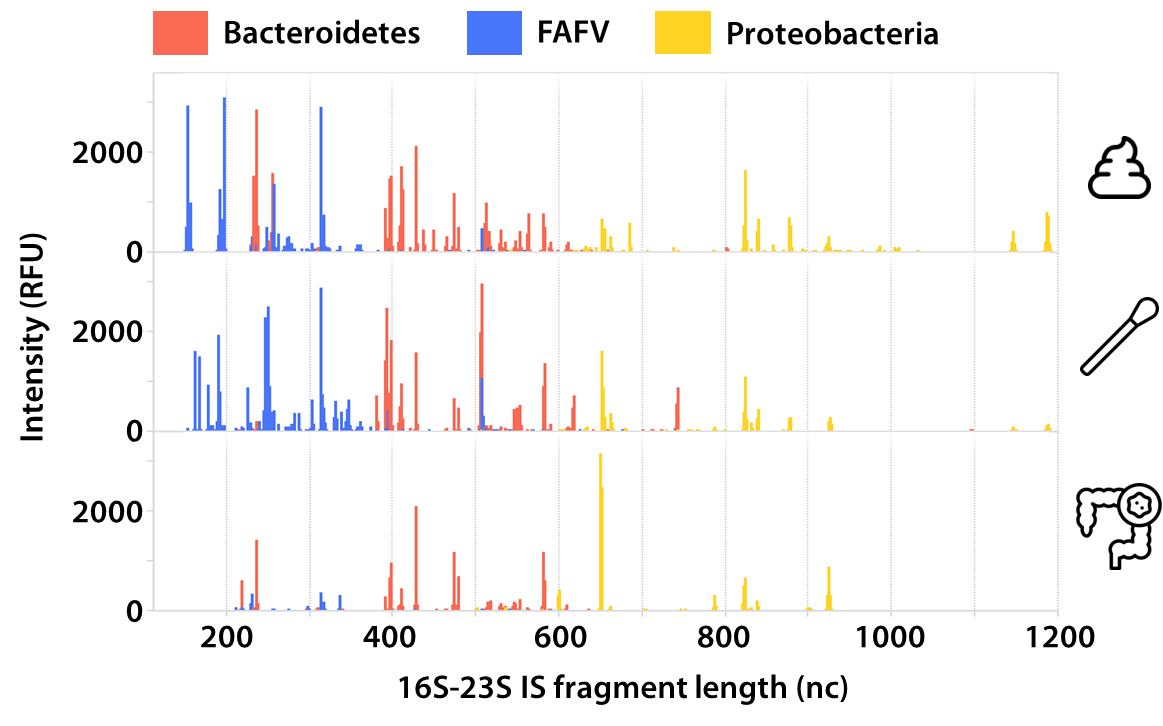

**Figure S1.** Example of microbiota profiles of a faecal sample, rectal swab, and mucosal biopsy of a single patient. X-axis: 16S-23S interspace (IS) fragment length in nucleotides. Y-axis: intensity in relative fluorescent units (RFU). The peaks within the same colour identify the specific species within that phylum. The position (nucleotide length) of each peak corresponds to a specific bacterial species with a certain intensity (abundance). *FAFV: Firmicutes, Actinobacteria, Fusobacteria, Verrucomicrobia.*

**
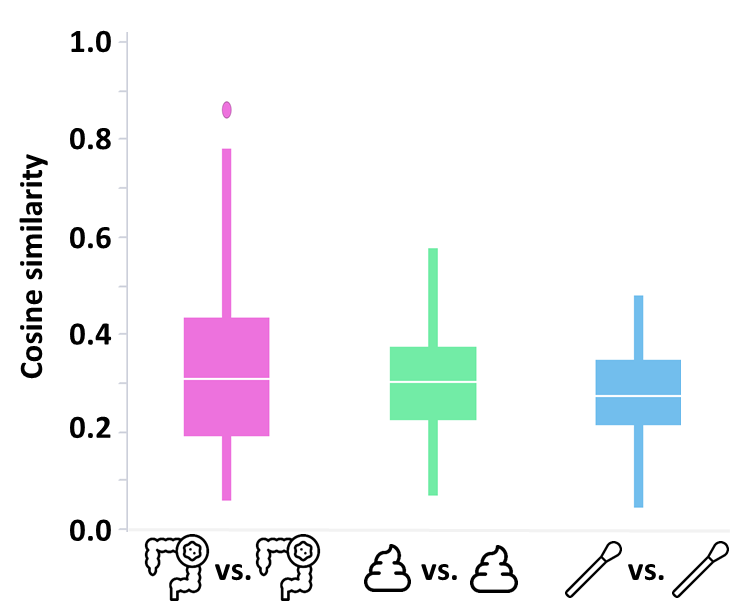
**
**Figure S2.** Cosine similarity between microbiota profiles of samples of the same sample type from different patients (colonic biopsies, n=190, fecal samples, n=175, or rectal swabs, n=153). The cosine similarity can range between 0 (completely different microbiota profiles) and 1 (identical profiles). The median and interquartile ranges of these similarities are visualized as boxplots.


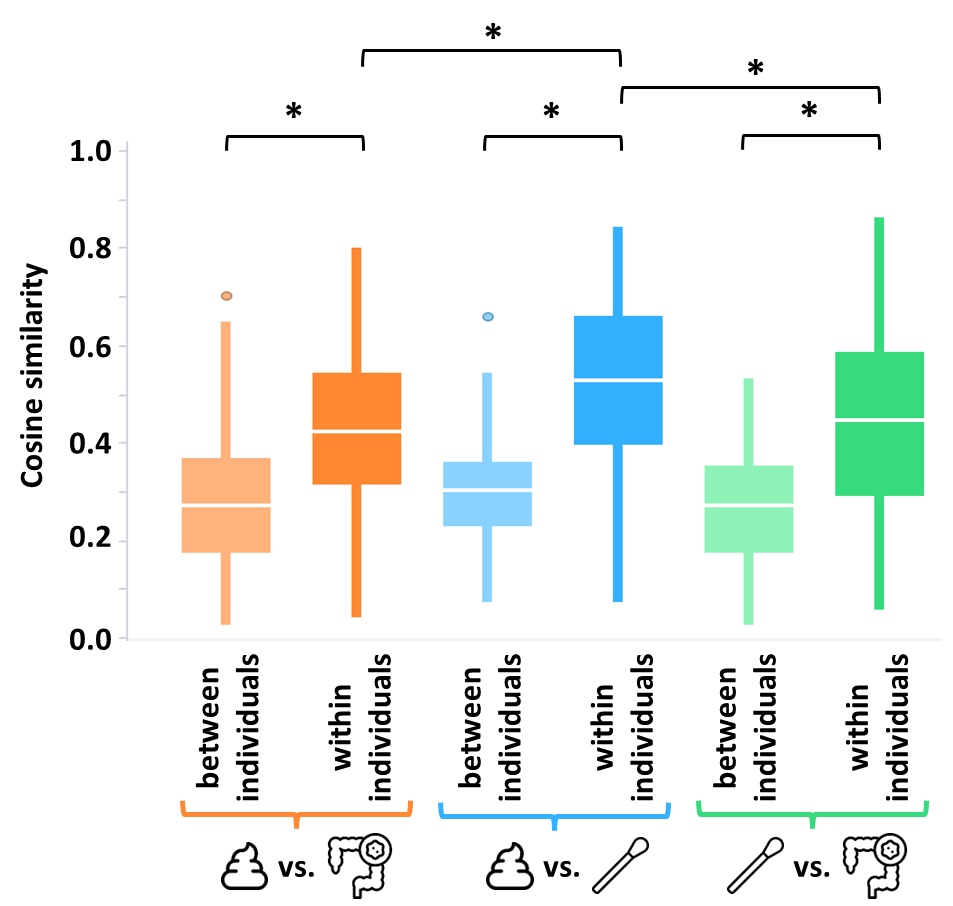

**Figure S3.** Cosine similarity between microbiota profiles of different sample types from different patients and within the same patient. **p<0.05*

**
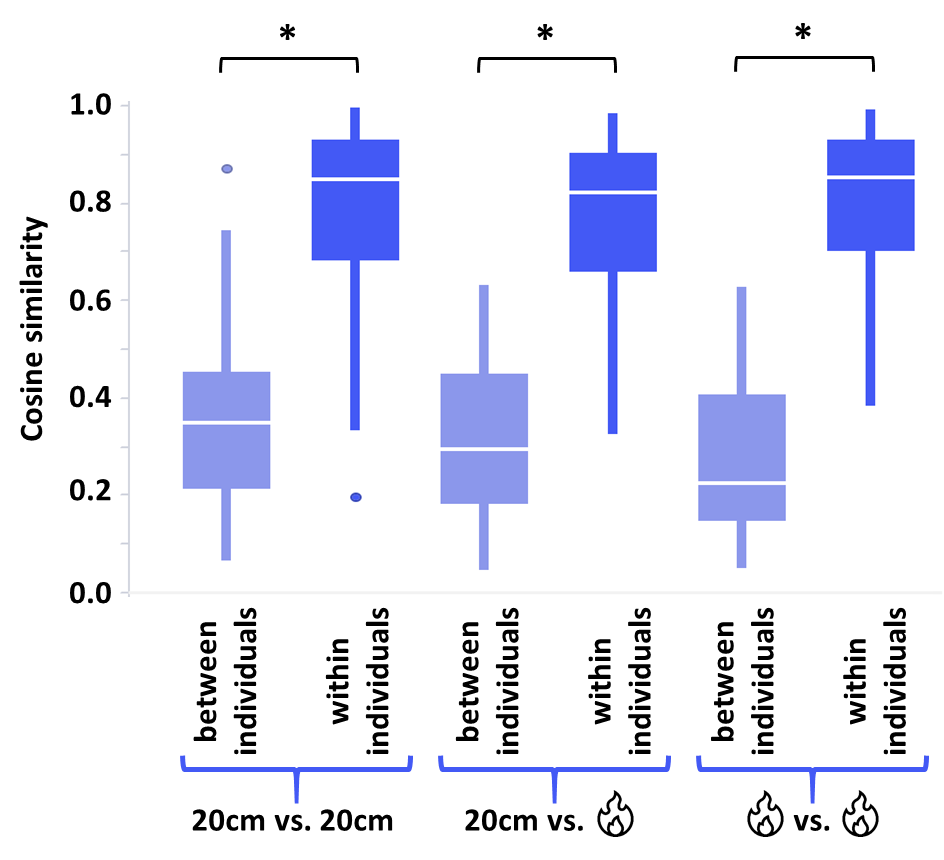

Figure S4.** Cosine similarity between microbiota profiles of different biopsy locations from different patients and within the same patient. *20cm: biopsy 20cm ab ano; fire icon: biopsy from most diseased bowel segment or an ulcer; *p<0.05.*

| **Comparison** | **Between/within individuals** | **Number of samples** | **Cosine similarity** *(median)* |
| --- | --- | --- | --- |
| *Identical sample types* |  |  |  |
| Biopsy vs. biopsy | between | 190 | 0.31, IQR [0.30-0.35] |
| Faeces vs. faeces | between | 175 | 0.31, IQR [0.29-0.33] |
| Rectal swab vs. rectal swab | between | 153 | 0.28, IQR [0.27-0.29] |
|  |  |  |  |
| *Different sample types* |  |  |  |
| Faeces vs. biopsy | between | 167 | 0.27, IQR [0.17-0.37] |
| Faeces vs. biopsy | within | 154 | 0.43, IQR [0.32-0.55] |
| Faeces vs. rectal swab | between | 150 | 0.30, IQR [0.23-0.36] |
| Faeces vs. rectal swab | within | 136 | 0.53, IQR [0.39-0.66] |
| Rectal swab vs. biopsy | between | 146 | 0.27, IQR [0.18-0.35] |
| Rectal swab vs. biopsy | within | 135 | 0.45, IQR [0.29-0.59] |
|  |  |  |  |
| *Different biopsy locations* |  |  |  |
| 20 cm vs. 20 cm | between | 167 | 0.35, IQR [0.21-0.45] |
| 20 cm vs. 20 cm | within | 159 | 0.85, IQR [0.68-0.93] |
| 20 cm vs. most diseased bowel segment or ulcer | between | 46 | 0.30, IQR [0.18-0.45] |
| 20 cm vs. most diseased bowel segment or ulcer | within | 45 | 0.82, IQR [0.66-0.90] |
| most diseased bowel segment or ulcer vs. most  diseased bowel segment or ulcer | between | 45 | 0.22, IQR [0.15-0.41] |
| most diseased bowel segment or ulcer vs. most  diseased bowel segment or ulcer | within | 29 | 0.85, IQR [0.70-0.93] |

**Table S1.** Cosine similarities between microbiota profiles of sample types/biopsy locations between/within individuals. *IQR: interquartile range.*

| **Comparison** | **Between/ within individuals** | **Number of samples in subgroup** | **Cosine similarity in subgroup** *(median)* | **For comparison:  Cosine similarity in all patients** *(median)* |  |
| --- | --- | --- | --- | --- | --- |
| *Crohn’s disease* |  |  |  |  |  |
| Faeces vs. biopsy | between | 61 | 0.25, IQR [0.20-0.27] | 0.27, IQR [0.17-0.37] |  |
| Faeces vs. biopsy | within | 53 | 0.45, IQR [0.32-0.53] | 0.43, IQR [0.32-0.55] |  |
| Faeces vs. rectal swab | between | 61 | 0.29, IQR [0.24-0.34] | 0.30, IQR [0.23-0.36] |  |
| Faeces vs. rectal swab | within | 46 | 0.59, IQR [0.44-0.71] | 0.53, IQR [0.39-0.66] |  |
| Rectal swab vs. biopsy | between | 52 | 0.26, IQR [0.22-0.30] | 0.27, IQR [0.18-0.35] |  |
| Rectal swab vs. biopsy | within | 45 | 0.44, IQR [0.28-0.53] | 0.45, IQR [0.29-0.59] |  |
|  |  |  |  |  |  |
| *Ulcerative colitis* | |  |  |  |  |
| Faeces vs. biopsy | | between | 113 | 0.28, IQR [0.23-0.32] | 0.27, IQR [0.17-0.37] |
| Faeces vs. biopsy | | within | 101 | 0.42, IQR [0.31-0.56] | 0.43, IQR [0.32-0.55] |
| Faeces vs. rectal swab | | between | 113 | 0.30, IQR [0.27-0.34] | 0.30, IQR [0.23-0.36] |
| Faeces vs. rectal swab | | within | 90 | 0.49, IQR [0.37-0.64] | 0.53, IQR [0.39-0.66] |
| Rectal swab vs. biopsy | | between | 100 | 0.25, IQR [0.20-0.30] | 0.27, IQR [0.18-0.35] |
| Rectal swab vs. biopsy | | within | 90 | 0.45, IQR [0.30-0.61] | 0.45, IQR [0.29-0.59] |
|  | |  |  |  |  |
| *Active disease (Crohn’s disease)* | |  |  |  |  |
| Faeces vs. biopsy | | between | 47 | 0.24, IQR [0.18-0.27] | 0.27, IQR [0.17-0.37] |
| Faeces vs. biopsy | | within | 40 | 0.41, IQR [0.31-0.53] | 0.43, IQR [0.32-0.55] |
| Faeces vs. rectal swab | | between | 47 | 0.30, IQR [0.24-0.32] | 0.30, IQR [0.23-0.36] |
| Faeces vs. rectal swab | | within | 35 | 0.59, IQR [0.44-0.68] | 0.53, IQR [0.39-0.66] |
| Rectal swab vs. biopsy | | between | 40 | 0.23, IQR [0.20-0.27] | 0.27, IQR [0.18-0.35] |
| Rectal swab vs. biopsy | | within | 34 | 0.42, IQR [0.27-0.52] | 0.45, IQR [0.29-0.59] |
|  | |  |  |  |  |
| *Inactive disease (Crohn’s disease)* | |  |  |  |  |
| Faeces vs. biopsy | | between | 38 | 0.25, IQR [0.21-0.31] | 0.27, IQR [0.17-0.37] |
| Faeces vs. biopsy | | within | 33 | 0.46, IQR [0.34-0.56] | 0.43, IQR [0.32-0.55] |
| Faeces vs. rectal swab | | between | 38 | 0.29, IQR [0.25-0.33] | 0.30, IQR [0.23-0.36] |
| Faeces vs. rectal swab | | within | 24 | 0.59, IQR [0.48-0.71] | 0.53, IQR [0.39-0.66] |
| Rectal swab vs. biopsy | | between | 29 | 0.26, IQR [0.22-0.30] | 0.27, IQR [0.18-0.35] |
| Rectal swab vs. biopsy | | within | 25 | 0.48, IQR [0.28-0.53] | 0.45, IQR [0.29-0.59] |
|  | |  |  |  |  |
| *Active disease (Ulcerative colitis)* | |  |  |  |  |
| Faeces vs. biopsy | | between | 99 | 0.28, IQR [0.22-0.33] | 0.27, IQR [0.17-0.37] |
| Faeces vs. biopsy | | within | 91 | 0.43, IQR [0.34-0.56] | 0.43, IQR [0.32-0.55] |
| Faeces vs. rectal swab | | between | 99 | 0.30, IQR [0.27-0.34] | 0.30, IQR [0.23-0.36] |
| Faeces vs. rectal swab | | within | 78 | 0.49, IQR [0.38-0.64] | 0.53, IQR [0.39-0.66] |
| Rectal swab vs. biopsy | | between | 87 | 0.25, IQR [0.20-0.31] | 0.27, IQR [0.18-0.35] |
| Rectal swab vs. biopsy | | within | 81 | 0.45, IQR [0.32-0.61] | 0.45, IQR [0.29-0.59] |
|  | |  |  |  |  |
| *Inactive disease (Ulcerative colitis)* | |  |  |  |  |
| Faeces vs. biopsy | | between | 35 | 0.27, IQR [0.20-0.31] | 0.27, IQR [0.17-0.37] |
| Faeces vs. biopsy | | within | 29 | 0.42, IQR [0.24-0.55] | 0.43, IQR [0.32-0.55] |
| Faeces vs. rectal swab | | between | 35 | 0.30, IQR [0.29-0.35] | 0.30, IQR [0.23-0.36] |
| Faeces vs. rectal swab | | within | 31 | 0.53, IQR [0.36-0.69] | 0.53, IQR [0.39-0.66] |
| Rectal swab vs. biopsy | | between | 37 | 0.27, IQR [0.24-0.31] | 0.27, IQR [0.18-0.35] |
| Rectal swab vs. biopsy | | within | 31 | 0.48, IQR [0.30-0.63] | 0.45, IQR [0.29-0.59] |

**Table S2.** Stratified analyses for IBD type and for disease activity per IBD type. Cosine similarities between microbiota profiles of sample types/biopsy locations between/within individuals. *IQR: interquartile range.*
